# Fully Differentiated HIV-1 Specific CD8+ T Effector Cells Are More Frequently Detectable in Controlled than in Progressive HIV-1 Infection

**DOI:** 10.1371/journal.pone.0000321

**Published:** 2007-03-28

**Authors:** Marylyn M. Addo, Rika Draenert, Almas Rathod, Cori L. Verrill, Benjamin T. Davis, Rajesh T. Gandhi, Gregory K. Robbins, Nesli O. Basgoz, David R. Stone, Daniel E. Cohen, Mary N. Johnston, Theresa Flynn, Alysse G. Wurcel, Eric S. Rosenberg, Marcus Altfeld, Bruce D. Walker

**Affiliations:** 1 Partners AIDS Research Center and Infectious Disease Division, Massachusetts General Hospital and Harvard Medical School Division of AIDS, Boston, Massachusetts, United States of America; 2 Howard Hughes Medical Institute, Partners AIDS Research Center and Infectious Disease Division, Massachusetts General Hospital and Harvard Medical School Division of AIDS, Boston, Massachusetts, United States of America; 3 Lemuel Shattuck Hospital, Boston, Massachusetts, United States of America; 4 Fenway Community Health Center, Boston, Massachusetts, United States of America; AIDS Research Center, Chinese Academy of Medical Sciences and Peking Union Medical College, China

## Abstract

**Background:**

CD8+ T cells impact control of viral infections by direct elimination of infected cells and secretion of a number of soluble factors. In HIV-1 infection, persistent HIV-1 specific IFN-γ+ CD8+ T cell responses are detected in the setting of disease progression, consistent with functional impairment in vivo. Recent data suggest that impaired maturation, as defined by the lineage markers CD45RA and CCR7, may contribute to a lack of immune control by these responses.

**Methodology/Principal Findings:**

We investigated the maturation phenotype of epitope-specific CD8+ T cell responses directed against HIV-1 in 42 chronically infected, untreated individuals, 22 of whom were “Controllers” (median 1140 RNA copies/ml plasma, range<50 to 2520), and 20 “progressors” of whom had advanced disease and high viral loads (median 135,500 RNA copies/ml plasma, range 12100 to >750000). Evaluation of a mean of 5 epitopes per person revealed that terminally differentiated CD8+ T cells directed against HIV-1 are more often seen in HIV-1 Controllers (16/22; 73%) compared to HIV-1 progressors (7/20; 35%)(p = 0.015), but the maturation state of epitope-specific responses within a given individual was quite variable. Maturation phenotype was independent of the HLA restriction or the specificity of a given CD8+ T cell response and individual epitopes associated with slow disease progression were not more likely to be terminally differentiated.

**Conclusions/Significance:**

These data indicate that although full maturation of epitope-specific CD8+ T cell responses is associated with viral control, the maturation status of HIV-1 specific CD8+ T cell responses within a given individual are quite heterogeneous, suggesting epitope-specific influences on CD8+ T cell function.

## Introduction

An increasing body of evidence suggests that HIV-1 specific T cells contribute to viral control in HIV-1 infection. CD8+ T cell responses in particular are thought to be crucial for control of HIV-1 viremia by direct elimination of infected cells and secretion of a number of soluble factors, but ultimately fail to control viremia in most infected persons (reviewed in[1]–[3]). HIV-1 specific CD8+ T cells appear coincident with the initial drop in HIV-1 viral loads during primary HIV-infection, which suggests a role for these cells in containment of viral replication [4], [5]. Further evidence for the antiviral activity of + T cells is provided by the simian AIDS model, in which animals fail to contain initial viremia upon depletion of CD8+ cells at the time of infection [6], [7]. Moreover, growing evidence of viral escape within CD8+ T cell epitopes during acute and chronic SIV or HIV-1 infection demonstrate that CD8+ T cells can exert strong immune selection pressure on the virus [8]–[10], [11], [12], [13], although the evidence for ongoing selection pressure in chronic infection is less clear-cut [14].

Despite this strong evidence of an antiviral effect of CD8+ T cells, most infected persons experience progressive loss of CD4+ T cells and increase in plasma viremia and ultimately develop clinical AIDS. Recent data suggest that the breadth and magnitude of the CD8+ T cell response directed against HIV-1 as measured by IFN-γ production does not correlate with HIV-1 viral load [15]–[17]. Furthermore HIV-1 specific CD8+ T cells persist in high numbers in persons with untreated chronic progressive disease and no quantitative differences in the HIV-1 specific T cell response were observed between individuals with progressive and long-term non-progressive infection [14]–[16]. These findings suggest that CD8+ T cell characteristics that determine differences in HIV-1 disease outcomes may be of a qualitative rather than a quantitative nature.

Studies have demonstrated that HIV-1 specific CD8+ T cells are impaired in their cytolytic function in chronic progressive infection [18], [19], and it was suggested that the inability of virus-specific CD8+ T cells to control viremia in chronic HIV-1 infection may be linked to failure of these lymphocytes to fully mature into effector cell [20]. In particular, a block in the maturation of virus-specific CD8+ T cells from an effector memory phenotype to terminally differentiated effector cell has been proposed in chronic progressive HIV-1 infection [20]. We therefore hypothesized that individuals with exquisite long-term control of viral replication during untreated chronic HIV-1 infection would possess significantly higher numbers of terminally differentiated CD8+ T cells directed against HIV-1 compared to subjects with progressive disease.

To address this hypothesis we determined the HIV-1 specific CD8+ T cell response to the entire expressed HIV-1 proteome and investigated the differentiation phenotype of 253 HIV-1 specific T cell responses using the lineage markers CD45RA and CCR7 in two very distinct HIV-1 infected study populations: a cohort of 22 individuals who were able to maintain control of viremia (median 1140 RNA copies/ml, range<50–2520) without the need for antiretroviral therapy (HIV-1 Controllers), and 20 HIV-1 infected individuals with untreated progressive chronic HIV-1 infection and high plasma viral loads (HIV-1 progressors). Our results demonstrate that HIV-1-specific CD8+ T cells of mature effector phenotype are more frequently detectable in HIV-1 Controllers than in HIV-1 progressors, but that the maturation status of HIV-1 specific CD8+ T cell responses within a given individual is quite heterogeneous, suggesting epitope-specific influences on maturation status.

## Methods

### Study subjects

Forty-two individuals with chronic untreated HIV-1 infection were recruited from the Massachusetts General Hospital (MGH), the Lemuel Shattuck Hospital and the Fenway Community Health Center in Boston, USA. Twenty-two study subjects were asymptomatic treatment-naïve individuals with persistent long-term control (median 15 years, range 3–22) of HIV-1 viremia <3000 RNA copies/ml (Roche Amplicor Assay Version 1.0) in the absence of any antiretroviral therapy (“HIV-1 Controllers”). The median plasma viral load in these HIV-1 controllers was 1140 HIV-1 RNA copies/ml (range<50 to 2520) and the median CD4 T cell count was 664 cells/µl (range 440–1097). Twenty additional study subjects had chronic progressive HIV-1 infection and fulfilled at least one of three criteria: HIV-1 plasma viral load>45,000 copies/ml, CD4 count<350/µl or an AIDS defining illness (“HIV-1 Progressors”)[14]. Among the HIV-1 Progressors the plasma viral load ranged from 12,100 to >750,000 (median 135,500) HIV-1 RNA copies/ml and the median CD4 T cell count was 245 cells/µl (range 12–630). Both, viral loads and CD4+ T cell counts differed significantly between the two cohorts (p = 0.0005 and p<0.0001, respectively). Clinical and demographic characteristics of the study subjects are summarized in table 1. The study was approved by the Institutional Review Boards of the participating institutions and all individuals gave informed consent for participation in the studies.

**Table 1:** Clinical characteristics of study cohorts*.

Parameter	Controllers n = 22	Progressors n = 20
Viral load (copies/ml)	1140 (<50–2520)	135,500 (12,900–>750,000)
CD4 count (cells/µl)	664 (440–1722)	254 (12–630)
Age	42 (26–64)	45 (33–73)
Gender (M/F)	18/5	13/7
Race (cauc./non-cauc.)	16/7	15/5

M = male; F = female;

* Clinical and immunologic data for some of the HIV-1 progressors have previously been described [14], [15].

All study subjects were without antiretroviral treatment.

### Peptides

Peptides were synthesized using an automated peptide synthesizer (MBS 396; Advanced Chemtech, Louisville, USA) using fluorenylmethoxycarbonyl chemistry. Four-hundred and ten peptides (16 to 19 amino acids long, 10 amino acid overlap, consensus sequence clade B 2001 (http://hiv-web.lanl.gov)) spanning all expressed HIV-1 proteins (Gag, Nef, Rev, Tat, Vpu, Vpr, Pol, Env, Vif) were synthesized as well as 8 to 11mer peptides corresponding to HLA-matched optimal epitopes previously described in the literature [21]. Clade B consensus sequence was chosen as the large majority of individuals in Boston are infected with HIV-1 clade B.

### Elispot assay

HIV-1 specific CD8+ T cell responses were quantified by Elispot assay using fresh peripheral blood mononuclear cells (PBMC) (0.5–1×10^5^ per well) and single peptides (final concentration: 14 µg/ml), as described previously [14], [15]. Briefly, fresh PBMC were plated in 96-well polyvinylidene plates (Millipore, Bedford, Mass.) that had been precoated with 0.5 µg of anti-IFN-γ monoclonal antibody, 1-DIK (Mabtech, Stockholm, Sweden)/ml. PBMC were added at a concentration of 100,000 cells per well in a volume of 100 µl of R10 medium (RPMI 1640,Sigma; 10% fetal calf serum; Sigma, 10 mM HEPES buffer; Sigma) with antibiotics (2 mM L-glutamine, 50 U of penicillin-streptomycin/ml). The final concentration of the peptides in the well was 20 µg/ml. Plates were incubated overnight at 37°C, 5% CO2, and developed as described previously [22]. Wells containing PBMC and R10 medium were used as negative controls and were run in triplicate on each plate. IFN-γ-producing cells were counted by direct visualization on an automatic Elispotreader (AID Immundiagnostika, Germany) and are expressed as spot-forming cells (SFC) per 10^6^ PBMC. Negative controls were always </ = 30 SFC per 10^6^ input cells. Positive controls consisted of incubation of PBMC with phytohemagglutinin (PHA), and only assays with PBMC in which PHA induced a significant stimulation, as defined by an well that was entirely covered with spots (“black well”) were included in the analysis to eliminate false negative results from damaged PBMC. Wells were counted as positive if they were at least 60 SFC/10^6^ PBMC and at least three times above background [15]. Breadth and magnitude were determined as described previously [15].

### Flow-cytometric detection of antigen-induced intracellular cytokine secretion and maturation phenotype

Based on the full-proteome Elispot screening a median of 5 (range 3–12) immunodominant peptides per patient (defined as the strongest HIV-1-specific CD8+ T cell responses in a given individual) were selected for further flow-cytometry-based analysis. In a subset of experiments pools of overlapping peptides spanning the HIV-1 proteins Gag, Pol, Env and Nef were used as antigens. Intracellular staining assays were carried out as described previously [23], [24]. Briefly, frozen PBMC were incubated with 20 µl of peptide with PBMC and 1 µg/ml of anti-CD28 and anti-CD49d antibodies (BD Biosciences) were added. Cells were incubated for one hour at 37°C, 5% CO2, followed by an additional 5 hours in the presence of a secretion inhibitor (Brefeldin A, 10μg/ml, Sigma). Cells were then stained with surface antibodies (CD8, CCR7, CD45RA; BD Biosciences) at 4°C for 30 minutes. After two washes, cells were fixed and permeabilized using the Caltag Cell Fixation and Permeabilization kit according to the manufacturer's protocol (Caltag, Burlingame, CA). Subsequently, intracellular antigen staining was performed using 15 µl of anti-IFN-γ specific monoclonal antibodies (BD Biosciences). Cells were then washed and analyzed on a FACS Calibur flowcytometry instrument (BD Biosciences) using FITC, PE, PerCP and APC as fluorescent parameters. Control conditions were established by the use of autologous PBMC, which had not been stimulated with peptide, but otherwise had been treated identically. PBMC stimulated with Phorbol-12-myristat-13-acetat/Ionomycin (PMA/I) were used as positive controls, and only assays in which >10% of cells in the lymphocyte gate responded to PMA/I stimulation were included in the analysis to control for false negative results from damaged PBMC. An IFN-γ- response was considered positive if >0.03% and ≥3x the background IFN-γ production. An HIV-1 specific CD8+ T cell response with >20% of CD45RA+/CCR7- cells was considered to be of terminally differentiated effector phenotype in this study, as described previously [25].

### Statistical analysis

Data are indicated as median and range or means and standard deviations. Differences between nominal data were tested for statistical significance by use of the paired Wilcoxon rank sum test or Mann Whitney two-tailed t-test. A p-value of <0.05 was considered significant. Differences between categorical data were calculated using Fisher's exact test. Data analysis and graphical presentation were performed using the Graphpad Prism 4.0 software package.

## Results

### HIV-1 specific T cell responses do not differ in breadth and magnitude between HIV-1 Controllers and HIV-1 Progressors

In order to determine the exact epitopic regions targeted by HIV-1-specific CD8+ T cells in infected individuals, we first performed a full proteome screening for HIV-1 specific T cell responses by IFN-γ Elispot using 410 overlapping peptides spanning HIV-1 clade B in all study subjects. The median magnitude of the total HIV-1-specific response in HIV-1 Controllers was 9,110 SFC/10^6^ PBMC (range 1,259 to 50,100) compared to 5,756.5 SFC/10^6^ PBMC (range 710 to 21,380) in HIV-1 Progressors. HIV-1 Controllers recognized a median of 18 epitopic regions (range 4-51) compared to 15 in the Progressor cohort (range 1 to 39). In line with results in chronic HIV-1 infection [16], [17], [26], no statistical difference was observed between either breadth or magnitude of the HIV-1-specific T cell response as measured by IFN-γ-production in HIV-1 Controllers and Progressors (p = 0.5 and p = 0.21, respectively), as previously reported for a small subset of these study subjects [14], [15].

### Fully differentiated CD8+ T cells were more frequently detected in HIV-1 Controllers

Recent data suggest a model of differentiation from naïve to antigen-specific memory T cells based on the lineage markers CCR7, which is a lymph node homing receptor and the RA isoform of CD45, which is a marker expressed on naïve lymphocytes, and is re-expressed at the fully mature stage of differentiation. Using these two parameters, a differentiation block from effector memory cells (CCR7^−^/CCD45RA^−^) to terminally differentiated effector cells (CCR7^−^/CD45RA^+^) has been reported in chronic progressive HIV-1 infection, resulting in a relative paucity of fully mature virus-specific effector CD8+ T cells [20], [27]. Based on this model and the lack of quantitative differences in the HIV-1 specific cellular immune response measured by IFN-γ secretion described above [14], [26], we hypothesized that HIV-1 Controllers would posses significantly higher numbers of terminally differentiated CD8+ T cells in directly isolated PBMC compared to HIV-1 Progressors.

**Figure 1:**
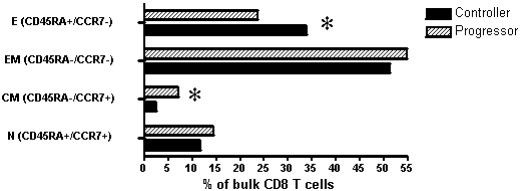
T cell differentiation profile in bulk CD8+ T cells. Based on the lineage markers, CD45RA and CCR7 bulk CD8+ T cells of HIV-1 Controllers (black bars) and HIV-1 progressors (hatched bars) were stratified into 4 subpopulations: effector (E), effector memory (EM), central memory (CM) and naïve (N) CD8+ T cells. Terminally differentiated (E) CD8+ T cells were of significantly higher frequency in HIV-1 Controllers (p = 0.017, Mann-Whitney), while Progressors had higher levels of CM cells (p = 0.02), indicated by asterisks (*).

To investigate this hypothesis, we examined maturation phenotype in unstimulated bulk CD8+ T cells from persons with progressive and controlled infection. Based on the expression of the markers CD45RA and CCR7, HIV-1 Controllers had a significantly higher frequency of fully differentiated effector CD8+ cells (CD45RA+/CCR7−) compared to HIV-1 Progressors (p = 0.02). In contrast, HIV-1 Progressors had higher mean frequencies of effector memory (CD45RA^−^/CCR7^−^), central memory (CD45RA^−^/CCR7^+^) and naïve T cells (CD45RA^+^/CCR7^+^) compared to HIV-1 Controllers, but these differences reached statistical significance only for the central memory cell subset (p = 0.03)(Figure 1a), and the lower proportion of central memory bulk CD8+ T cell subsets in the Controllers might be a reflection of the relative increase in the fraction of fully differentiated effector cells.

Given these results with unstimulated bulk CD8+ T cells, we next investigated the maturation phenotype of the HIV-1-specific fraction of CD8+ T cells. Four pools of overlapping peptides corresponding to the individual HIV-1 proteins Gag, Pol, Env and Nef, respectively, were used as antigenic stimuli for IFN-γ ICS assays in 10 randomly-selected individuals: five HIV-1 Controllers and 5 HIV-1 progressors. The use of peptide pools as antigenic stimulation did not allow us to detect differences in the differentiation status of HIV-1-specific CD8+ T cells between Controllers and Progressors (p = 0.44 Fisher's exact, data not shown).

### HIV-1-specific CD8+ T cell responses to different epitopes within the same HIV-1 protein differ in their differentiation status

**Figure 2:**
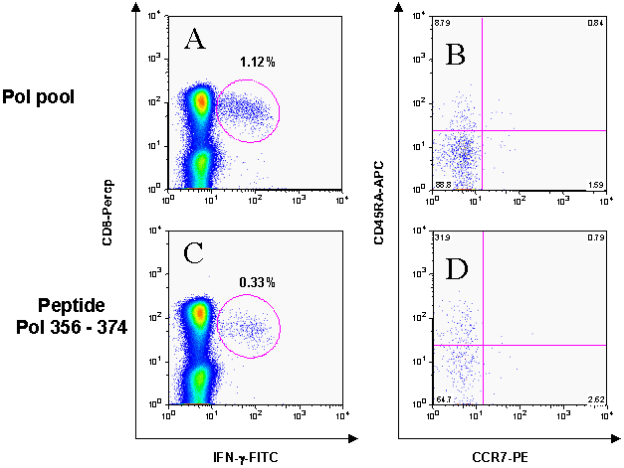
The effector phenotype of an individual CD8+T cell response may not be adequately reflected in a peptide pool. Panels A and C show IFN-γ production upon stimulation with the Pol peptide pool (A) and an individual Pol peptide (C). Panels B and D are gated on the IFN-γ producing cells and show differentiation phenotype of these HIV-1 specific cells as defined by CCR7 and CD45RA isoform. The terminally differentiated response to the Pol peptide 356–374 (31.9% CCR7−/CD45RA+ of gated cells) is diluted in the response to the entire Pol peptide pool (only 8.8% gated cells are CCR7−/CD45RA+).

The above-described approach using pools of overlapping peptides spanning individual HIV-1 proteins to stimulate HIV-1-specific CD8+ T cell responses did not allow one to differentiate among multiple epitope-specific responses to individual peptides in the pool, as the potential presence of phenotypic heterogeneity would obscure detection of differences. We therefore compared results using the 4 peptide pools to results obtained using individual overlapping peptides corresponding to specific immunodominant CD8+ T cell responses within the same protein in a subset of eight individuals (4 Controllers, 4 Progressors). In 5/8 (62%) individuals, a single fully mature response to an HIV-1 specific epitope was not reflected in the peptide pool corresponding to the protein, which as a whole showed <20% CD45RA+/CCR7− protein-specific CD8+ T cells. Figure 2 shows that for a representative study subject, within a single individual, the CD8+ T cell response to the same protein antigen, in this case Pol, can include both terminally differentiated CD8+ T cell responses to one epitope (Pol 356–374, figure 2 C and D) and non-terminally differentiated CD8+ T cell responses to one or more other epitopes, as reflected by the entire Pol peptide pool (Fig 2A and B). Overall, the single mature CD8+ T cell response to peptide Pol 356–374 (32% CD45RA+CCR7− CD8+ T cells) was diluted in the cells stimulated by the entire Pol pool, which as a whole does not reflect a fully mature phenotype (8.8% CD45RA+CCR7− CD8+ T cells). Given these data demonstrating that the use of peptide pools may obscure differences in maturation phenotype on the single epitope level, we extended the examination of the maturation status of individual epitope-specific CD8+ T cell responses to the total study population of 44 subjects.

### Detailed maturation phenotype analysis on the single epitope level reveals higher frequencies of terminally differentiated CD8+ T cells in HIV-1 Controllers

We chose the detailed single epitope analysis outlined above to evaluate a total of 126 immunodominant HIV-1 specific CD8+ T cell responses in the HIV-1 Controllers (median: 5, range 3–12 per individual) and 106 responses in the HIV-1 progressors (median: 5, range 1–12) for expression of CCR7 and CD45RA (note that for the one individual with only one investigated CD8+ response, this response represented the only detectable HIV-1 specific T cell response, in all other subjects at least 3 responses were analyzed). By using peptides that were previously shown to be recognized by CD8+ T cell responses in the respective study subjects, we were able to test both immunodominant T cell epitopes that had not been optimally defined as well as epitopes restricted by HLA alleles for which tetramers were not readily available.

**Figure 3:**
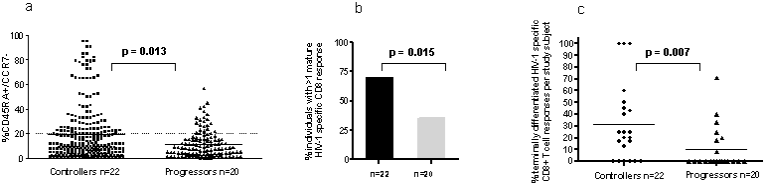
Terminally differentiated HIV-1-speficic CD8+ T cells are more frequently detectable in HIV-1 Controllers compared to HIV-1 Progressors. (A) Percentage of CD45RA+/CCR7−/CD8+ T cells for the 126 and 106 HIV-1-specific CD8+ T cell responses tested in HIV-1 Controllers and Progressors, respectively. Frequencies of effector phenotype T cell responses were significantly higher in HIV-1 Controllers (squares) compared to progressors (triangles). An HIV-1 specific CD8+ T cell response with >20% of CD45RA+/CCR7− cells was considered to be of terminally differentiated phenotype (dotted line) [25]. (B) More HIV-1 Controllers had at least one terminally differentiated HIV-1 specific T cell response compared to HIV-1 Progressors (p = 0.015, Fisher's exact). (C) Of the CD8+ T cell responses against HIV-1 tested per individual a higher percentage of terminally differentiated responses were detectable in HIV-1 Controllers (p = 0.007, Mann Whitney). For 3 Controllers all investigated responses were of the terminally differentiated phenotype, while five Controllers had none.

As depicted in figure 3a, flow-cytometry-based investigation of all CD8+ T cell responses tested for maturation phenotype in the two study populations revealed that the median percentage of terminally differentiated CD45RA^+^/CCR7^−^ HIV-1 specific CD8+ T cell responses was significantly different between HIV-1 Controllers and HIV-1 Progressors (p = 0.013). In addition, 16/22 (73%) of HIV-1 Controllers had at least one terminally differentiated HIV-1 specific CD8+ T cell response, while responses of mature effector phenotype could only be found in 7/20 (35%) of the HIV-1 Progressors (p = 0.015, Fisher's exact test)(Figure 3b).

In a subsequent analysis we determined the percentage of terminally differentiated CD8+ T cell responses per individual. In the HIV-1 Controllers a mean of 32% of all responses tested were of the mature effector phenotype, while only a mean of 11% of tested responses was terminally differentiated in HIV-1 progressors (p = 0.007, Mann Whitney test) (Figure 3c). For three HIV-1 Controllers, all tested epitopes demonstrated a mature effector phenotype, a phenomenon that was not observed in any of the HIV-1 progressors. However, the percentages of fully differentiated HIV-1 specific T cell responses displayed a wide range (0%–100% in HIV-1-Controllers and 0%–71% in HIV-1 progressors) (Figure 3c).

Taken together, these data show that terminally differentiated CD8+ T cell responses are more readily detectable in HIV-1 Controllers than in HIV-1 Progressors, demonstrating a qualitative difference in CD8+ T cell differentiation between controlled and progressive chronic HIV-1 infection.

### Differentiation phenotype of a given epitope is independent of HLA type and epitope specificity

**Figure 4:**
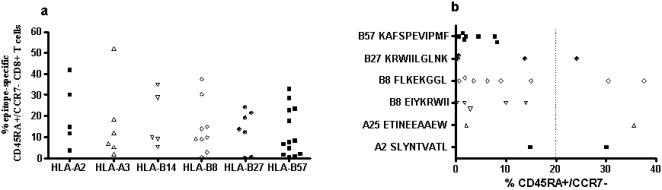
Maturation phenotype of HIV-1 specific CD8+ T cells by HLA type and epitope specificity. Panel A shows the percentage of CD45RA+/CCR7− CD8+ T cells of 46 HLA-A2, -A3, -B14, -B27 and -B57-restricted epitopes tested in the study cohort. Panel B depicts the percentage of CD45RA+/CCR7− CD8+ T cells specific for six specific HIV-1 epitopes tested (restricting HLA allele and peptide sequence are shown). Data reflective of a total of 21 study subjects, who had responses to the specific HLA-matched optimal epitopes tested (14 Controllers and 5 Progressors).

Based on the large number of datapoints on the CD8+ T cell phenotype of individual peptide-specific T cell responses described above we evaluated potential predictive factors for the presence of terminally differentiated responses, including the restricting HLA class I allele and the specific targeted epitopes. We first investigated if HIV-1 CD8+ T cell epitopes restricted by specific HLA class I alleles were more likely to express CD45RA+/CCR7− by testing a total of 45 optimal epitopes restricted by HLA alleles frequently encountered in this study cohort (HLA-A2, -A3, -B8, -B14, -B27 and -B57). As depicted in Figure 4a, there was no clear association between the HIV-1-specific CD8+ T cell effector phenotype and the restriction by specific HLA alleles. For example, HLA-A2-restricted epitopes were terminally differentiated in some individuals, but not in others, and different HLA-A-restricted epitopes within one individual study subject displayed different maturation phenotypes.

In addition, we investigated whether CD8+ T cells targeting epitopes restricted by HLA-B27 and -B57, two alleles that have been associated with lower viral setpoints and long-term non-progressive HIV-1 infection, were predominantly of a terminally differentiated phenotype [28]–[30] and found this not to be the case (Fig 4b). For example, CD8+ T cell responses directed against the HLA-B57-restricted epitope KAFSPEVIPMF, an epitope recognized by the vast majority of HLA-B57 positive individuals, did not show a mature effector phenotype in any of the study subjects. In contrast, the HLA-B27-restricted epitope KRWIILGLNK, for which there is strong evidence in the literature that viral escape and loss of recognition of the epitope leads to disease progression [28]–[30], had variable maturation phenotypes in different study subjects despite the fact that all individuals tested for this epitope were HIV-1 Controllers with comparable viral loads. Furthermore, CD8+ T cell responses directed against the same HIV-1 epitope exhibited variable maturation phenotypes in different individuals irrespective of Controller or Progressor disease status (Fig. 4b).

Taken together, these data suggest that maturation phenotype of a given T cell response is not predictable by the restricting HLA type or by the epitope specificity. This indicates that although full maturation of CD8+ T cells clearly seems to occur more frequently in HIV-1 Controllers, epitope-specific CD8+ T cells can vary in their phenotype within a given individual, as do CD8+ T cell responses specific for the same epitope between different individuals.

## Discussion

Recent studies indicate that the functional characteristics of virus-specific T cells may be associated with the ability to control viral replication [26], [31]. We therefore performed a comprehensive analysis of HIV-1 specific CD8+ T cell responses in untreated HIV-1 infected individuals with progressive and non-progressive disease. No differences between these two very distinct patient populations were observed in terms of the magnitude and breadth of HIV-1-specific CD8+ T cell responses as measured by antigen-specific IFN-γ production, consistent with previous reports [15]–[17]. In contrast, terminally differentiated CD45RA^+^/CCR7^−^ HIV-1-specific CD8+ T cells were significantly more frequently detectable in HIV-1 Controllers than in HIV-1 progressors, and contributed an average of 32% to the total HIV-1-specific CD8+ T cell response in HIV-1-Controllers, compared to only 11% in progressors. These data indicate that qualitative rather than quantitative characteristics of HIV-1-specific CD8+ T cell responses may be associated with differences in viral control and HIV-1 disease outcome.

Based on the linear differentiation model of CD8+ T cells defined by the lineage markers CD45RA and CCR7, it has been proposed that chronic progressive HIV-1 infection is related to a maturation block from effector memory phenotype (EM, CD45RA^−^/CCR7^−^) to full effector phenotype (E, CD45RA^+^/CCR7^−^)[20], [27]. Our data in HIV-1 Controllers compared to Progressors support this hypothesis, and are in line with recent studies limited to a few epitopes by Hess et al. who used identical differentiation markers demonstrating terminally differentiated HIV-1 specific CD8+ T cells in 6 subjects with non-progressive disease [25], and that early treatment of acute infection enhanced the detection of such fully differentiated responses. Chen et al. [19] compared EBV and HIV-1 specific CD8+ T cell responses in a small number of subjects and demonstrated that HIV-1 specific T cells in chronic HIV-1 infection lack CD45RA, also concordant with the data presented in this study. The linear differentiation model based on CD45RA and CCR7 as proposed by Champagne et al. [20] and other groups is only one of the various models of human T cell differentiation that have been proposed. However, studies based on other differentiation markers such as CD27 revealed similar findings in that levels of HIV-1 specific T cells exhibiting the CD8+/CD27− effector phenotype, which may largely overlap with the CD45RA+/CCR7− phenotype of the above quoted model, were associated with delayed disease progression in untreated HIV-1 infection [32], [33].

The present study represents an important extension beyond the scope of previous studies of HIV-1 specific T cell maturation, which were limited by small patient numbers and/or the utilization of MHC class I tetramers for the identification of HIV-1 specific T cells, which restrict the number, HLA types and targeted epitopes that can be tested in a given individual. Most reported studies have investigated at most 1–3 tetramers per patient in populations that had to be selected for HLA class I molecules and epitopes for which tetramers were readily available (commonly HLA-A2 and HLA-B8)[19], [25], [33]. In most cases these responses may only reflect a small fraction of the total HIV-1 specific response and selection for a narrow spectrum of HLA types may add an additional bias to the interpretation of data on a broader scale. Our simultaneous evaluation of multiple epitope-specific responses representing the dominant virus-specific T cell responses as determined by IFN-γ revealed heretofore unappreciated epitope-specific differences in CD8+ T cell differentiation, comparing very distinct study populations at the extremes of viral control. However, the use of peptides corresponding to HIV-1 clade B consensus sequences, and not to the autologous virus sequence in the individual study subject may still have resulted in the underestimation of the total virus-specific T cell response.

Not all epitopes in HIV-1 Controllers were of the effector phenotype, and previously defined epitopes such as HLA-A2-SLYNTVATL, which was investigated in all studies quoted above, exhibited different effector phenotypes in different individuals irrespective of controller or progressor status (Fig. 4). It is of particular interest to note that T cell responses restricted by HLA alleles associated with HIV-1 long-term nonprogression such as HLA-B27 or HLA-B57 epitopes did not uniformly display terminally differentiated maturation status. In particular, in none of our study subjects did we observe terminally differentiated CD8+ T cell response against the HLA-B57-restricted epitope KAFSPEVIPMF, which the vast majority of individuals expressing HLA-B57 recognize. Interestingly, this epitope, unlike other HLA-B57-restricted epitopes, is never found to exhibit sequence variation as a consequence of viral escape in previous studies [34]. This finding gives rise to the hypothesis that immature T cells lacking effector phenotype are less likely to exert immune selection pressure on the virus, a hypothesis that will need to be tested in future studies.

The observations outlined in this study raise the question as to whether terminally differentiated HIV-1 specific effector cells actually mediate viral control or if the setting of viral control subsequently allows for full CD8+ T cell maturation. In some studies addressing the functional properties of T cells such as proliferation or direct cytotoxicity, no clear association with effector phenotype defined by CCR7 and CD45RA could be identified [26]. However, other studies have reported higher granzyme and perforin content, elevated IFN-γ production and stronger direct cytolytic activity in CD8+ T cells with effector phenotype. The exact association of maturation phenotype and viral control may be much more complex and differentiated than suggested by previous studies and future studies combining full HIV-1 genome screening and the simultaneous use of a wide panel of differentiation and functional markers facilitated by the advent of high throughput multicolor flow-cytometry technology will aid in dissecting the relationship between maturation status of CD8+ T cells and viral control.

In conclusion, we show that fully differentiated CD8+ T cells specific for HIV-1 are more frequently detectable in individuals with exquisite control of HIV-1 infection and that impaired effector cell maturation may contribute to chronic progressive disease. Our data support the notion that the capacity for complete differentiation of HIV-1 specific T cells is associated with improved HIV-1 control and that phenotypic analysis is a useful adjunct in the comprehensive assessment of virus-specific T cells. However, differentiation phenotype is not predictable by HLA type or epitope specificity, which indicates that the exact association of maturation phenotype and viral control may be much more complex and differentiated than previously appreciated. Future studies combining full HIV-1 proteome CD4^+^ and CD8+ T cell screening and the simultaneous use of a wide panel of differentiation and functional markers will aid in dissecting the complexities of determining the exact properties of T cells needed to confer viral control, which should facilitate the rational design of new immunological and pharmaceutical approaches to viral control.
